# Exercise-based cardiac rehabilitation in patients with chronic heart failure: a Dutch practice guideline

**DOI:** 10.1007/s12471-014-0612-2

**Published:** 2014-12-10

**Authors:** R. J. Achttien, J. B. Staal, S. van der Voort, H. M. Kemps, H. Koers, M. W. A. Jongert, E. J. M. Hendriks, on behalf of the Practice Recommendations Development Group

**Affiliations:** 1 Scientific Institute for Quality of Healthcare, Radboud University Nijmegen Medical Centre, Geert Grooteplein 21 6500, PO Box 9101, Nijmegen, HB the Netherlands; 2 Rehabilitation Department Tergooiziekenhuizen, Zonnestraal, Hilversum, the Netherlands; 3 Department of Cardiology, Maxima Medical Center, Veldhoven; Department of Medical Informatics, Amsterdam Academic Medical Center, University of Amsterdam, Amsterdam, the Netherlands; 4 Hart op Koers, Gouda, the Netherlands; 5 Dutch Institute of Allied Health Care, Amersfoort, the Netherlands, The Hague University of Applied Sciences, The Hague, the Netherlands

**Keywords:** Chronic heart failure, Exercise-based, Guideline, Clinical practice

## Abstract

**Rationale:**

To improve the quality of exercise-based cardiac rehabilitation (CR) in patients with chronic heart failure (CHF) a practice guideline from the Dutch Royal Society for Physiotherapy (KNGF) has been developed.

**Guideline development:**

A systematic literature search was performed to formulate conclusions on the efficacy of exercise-based intervention during all CR phases in patients with CHF. Evidence was graded (1–4) according the Dutch evidence-based guideline development criteria.

**Clinical and research recommendations:**

Recommendations for exercise-based CR were formulated covering the following topics: mobilisation and treatment of pulmonary symptoms (if necessary) during the clinical phase, aerobic exercise, strength training (inspiratory muscle training and peripheral muscle training) and relaxation therapy during the outpatient CR phase, and adoption and monitoring training after outpatient CR.

**Applicability and implementation issues:**

This guideline provides the physiotherapist with an evidence-based instrument to assist in clinical decision-making regarding patients with CHF. The implementation of the guideline in clinical practice needs further evaluation.

**Conclusion:**

This guideline outlines best practice standards for physiotherapists concerning exercise-based CR in CHF patients. Research is needed on strategies to improve monitoring and follow-up of the maintenance of a physical active lifestyle after supervised CR.

## Rationale

Chronic heart failure (CHF) is defined as ‘a complex of signs and symptoms associated with a structural or functional abnormality of the heart’. [1] CHF involves peripheral and central changes, which are functional (as a compensation mechanism) in the short term, but have adverse consequences in the long term, resulting in reduced exercise capacity. The most frequent causes of CHF are hypertension and coronary artery disease; less frequent causes include heart valve diseases, arrhythmias and viral infections. The prevalence and incidence of CHF increases with age, and has an adverse overall prognosis with a 5-year mortality rate of 45 %. In 2012, 4136 women and 2625 men died as a consequence of CHF in the Netherlands [2].

Multidisciplinary cardiac rehabilitation (CR) reduces mortality and early hospital readmission, progressive deterioration of CHF and prevents recurrence of cardiac events. [3, 4] Exercise training, usually conducted by physiotherapists, constitutes an important part of CR aiming to improve exercise capacity and quality of life (QoL) both in the short and long term. The mechanisms underlying these beneficial effects involve improvement of muscle perfusion, muscle metabolism, ventilatory efficiency, neurohormonal regulation and cardiac function [4–6].

The exact content of exercise-based CR programs in CHF patients in the Netherlands is not well established. Within Dutch CR centres, there is considerable variation in the methods for determination of exercise intensity training, training intensity and volume. [7] A possible explanation is that guidelines and position statements lack clear practical guidance for physiotherapists. [8–14] Therefore, a clinical practice guideline on exercise-based CR was developed by the Dutch Royal Society for Physiotherapy (KNGF), describing optimal exercise-based CR, including assessment, treatment and evaluation in CHF patients. This clinical practical guideline and the guideline for exercise-based CR in patients with coronary artery disease [15] can be considered as a supplement to the Dutch Multidisciplinary Guideline for CR [9].

## Guideline development process

This guideline has been systematically developed according to the method of *Physiotherapy Development in the Netherlands*, [16] which is in line with international methods of guideline development [17].

A computerised literature search was undertaken in the Cochrane library, Medline, PEDro-database, Cinahl and relevant available national and international guidelines of CR, [8–14, 18] using the following key words (in Dutch and English): heart disease, chronic heart failure, systolic heart failure, congestive heart failure, treatment outcome, diagnosis, exercise, and physiotherapy.

Recommendations for exercise-based CR were, if they existed, based on systematic reviews or meta-analyses, and if available completed with more recent randomised-controlled trials (RCTs) and otherwise based on RCTs only. Methodological quality of RCTs was scored using the physiotherapy evidence database (PEDro) scale. [19] Only studies with a score of more than 5 out of 10 points were included. If there was insufficient scientific evidence, recommendations were based on consensus within the guideline development group (GDG).

The level of evidence was categorised on the basis of Dutch national agreements on evidence grading for guideline development (EBRO/CBO) (Table 1).

**Table 1:** Levels of scientific evidence

Level of evidence	Quality levels (intervention and prevention)
Level 1: study at A1 level or at least two independent A2 level studies	A1 Systematic review of at least two independent A2 level studies A2 Randomised, double-blind, comparative clinical trial of good quality and sufficient sample size
Level 2: one study at A2 level or at least two independent B level studies	B Comparative study not meeting all criteria mentioned under A2 (including case-control studies and cohort studies)
Level 3: one B or C level study	C Non-comparative study
Level 4: expert opinion^a^	D Opinions of experts, for instance the members of the guideline development team

^a^Additionally, other aspects were used to determine recommendations in this case, such as clinical relevance, safety, patient and professional perspective, availability of devices and resources, health organisations, and ethnical and organisational aspects

### Comments, modification and financing

The guideline is written by the GDG, consisting of the following disciplines: physiotherapists representing the KNGF, movement scientists, epidemiologists, a representative of the Dutch multidisciplinary CR guideline committee and a cardiologist representing the CR section of the Dutch Society of Cardiology. An external group, consisting of a clinical exercise physiologist, a physician, and two physiotherapists, reviewed the draft versions of the guideline. The members of the guideline GDG and the external members have declared that they have no conflict of interest. This study was funded by the KNGF.

## Clinical and research recommendations

The CR process is divided into the following phases:

- Clinical phase (phase I)
- Outpatient CR phase (phase II)
- Post-CR phase (Phase III)

This guideline focuses mainly on the outpatient CR phase (phase II).

### Clinical phase (phase I)

It should be noticed that the majority of patients with CHF are referred to CR straight from the outpatient setting, without recent clinical admission. This subset includes stable patients who remain symptomatic despite optimal medical and device therapy.

#### Recommendation 1. Stay at intensive care unit (ICU) or coronary care unit (CCU) and mobilisation during the clinical phase (phase I)

Relative rest is recommended during the patients’ stay at the CCU after an acute cardiac event or after their stay at the ICU following heart surgery. Dynamic mobilisation exercises and treatment of pulmonary symptoms (if necessary) results in a faster recovery and a better physical health at discharge in CHF patients undergoing revascularisation surgery [20, 21] (level 1), valve replacement and (left) ventricular surgery, and after decompensation or other cardiac events (level 4).

Postoperative pulmonary complications (such as obstructive pulmonary diseases) are treated if necessary (as indicated by the pulmonologist or other medical specialist) at the CCU or ICU. Perioperative treatment involves teaching the patient techniques to improve ventilation and to mobilise and cough up sputum (breathing, huffing and coughing techniques) and advising the patient.

The clinical mobilisation should include functional exercises, such as exercises related to activities of daily living (ADL) and walking at an early stage of this phase. Exercise should be discontinued or intensity should be decreased if patients show signs of excessive strain/cardiac overload. The physiotherapist explains the aetiology and/or the treatment (e.g. medication, surgery), ways of coping with CHF and other complaints during daily life (i.e. how to ‘respond to the demands of life’, and how to recognise signs of excessive strain), and how to gradually increase the intensity of activities at home. Table 2 lists the referral information provided before the mobilisation starts, signs of excessive strain/ cardiac overload and outcome criteria.

**Table 2:** Referral information and reasons to terminate training during mobilisation, and final outcome criteria (phase I)

Referral information	Reasons to terminate training	Outcome criteria^a^
Reason for referral	Angina	Able to function at a sufficient ADL level (including e.g. walking and self-care, with assistance if necessary)
Date of hospital admission	Impaired pump function (shortness of breath disproportionate to exertion, abnormal fatigue disproportionate to exertion, increased peripheral / central oedema)	At least some knowledge of their CHF, and if applicable about the treatment such as surgery (sternotomy, wound recovery etc.)
Diagnosis	Arrhythmias (high heart rate not in proportion to exertion, irregular heartbeat, changes in known arrhythmias)	The patient knows how to cope with their CHF symptoms and is able to increase exercise intensity and expand their ADL activities
Date of event or treatment	Abnormal increase or decrease of blood pressure	
Medication use (type and dosage regime)	Fainting	
Complications and/ or comorbidities	Dizziness	
Further diagnostic information deemed relevant by the cardiologist	Vegetative reactions (excessive perspiring, pallor)	

*ADL* activities of daily living, *CHF* chronic heart failure

^a^In some exceptional cases, patients may not have met these goals at the time of discharge from hospital, due to psychosomatic, social or severe physical problems (e.g. comorbidities). These patients may be referred for clinical admission to a specialised multidisciplinary CR centre for more intensive care

### Outpatient CR phase (phase II)

Patients with CHF will be referred to the CR team by their cardiologist when they have returned to a stable state (in terms of filling volume, medication use and functional classification) after a clinical admission or after a routine outpatient check-up. The outpatient CR consists of an intake / assessment procedure, a treatment phase and an evaluation, which will be discussed chronologically in the following sections.

#### Intake / assessment procedure

**Fig. 1:**
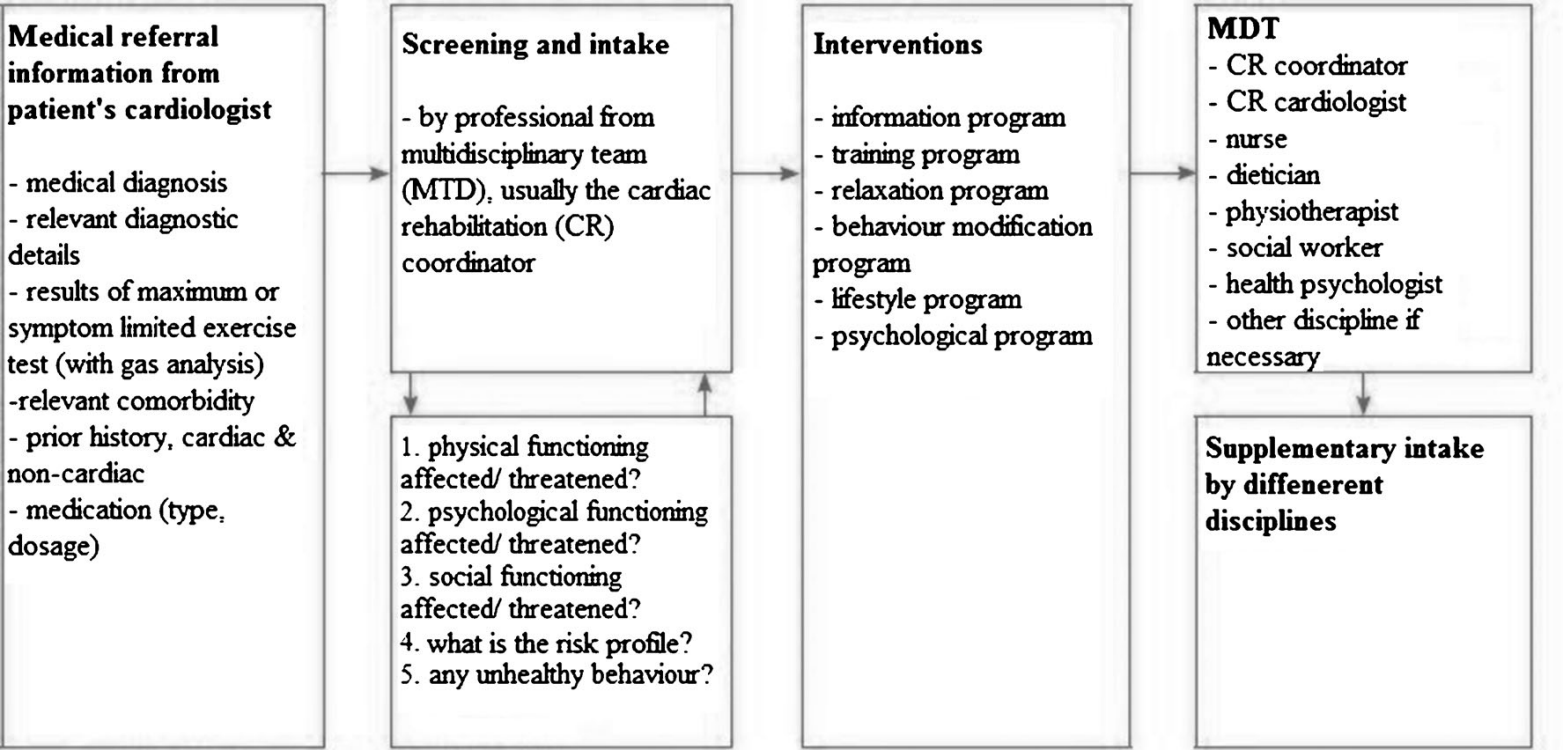
Flowchart of multidisciplinary cardiac rehabilitation screening

At the start of the outpatient CR phase, all eligible patients should be referred for an intake procedure, carried out by a member of the CR team, in many cases the CR coordinator/nurse, preferably by using a clinical algorithm for patient needs in CR (Fig. 1) [22].

Based on the results of the assessment procedure, the CR coordinator/nurse, in consultation with the patient, decides what type of care or what interventions are indicated. The patient then goes through a specific assessment for each of the relevant disciplines, after which they start one or more CR programs (Fig. 1.).

If the patient has no contraindications for physical training (Table 3), an additional assessment should be performed by the physiotherapist to define the content of the training program.

**Table 3:** Contraindications for training, signs of excessive strain and safety criteria (phase II)

Contraindications	Excessive strain	Safety criteria
• Progressive increase in heart failure symptoms	• Severe fatigue or dyspnoea out of proportion to the level of exertion	• ICD Cardiologist informs physiotherapist about safe heart rate range First 6–8 weeks after implantation no (submaximal) strength training of the upper extremities^a^
• Severe ischaemia of the cardiac muscle upon exertion	• Angina	
• Respiratory frequency of more than 30 breaths per minute	• Unexpected increase in breathing rate (>40 breaths per minute)	
• Dyspnoea while speaking	• Pulse pressure reduction (≥10 mmHg)	• Diabetes mellitus Check for wounds and sensory defects (monofilament test) Check blood glucose values before, during and after the exercise session. Blood glucose values ≤5 and ≥15 mmol/L are relative contraindications for exercising Retinopathy of grade ≥3 is a relative contraindication for training
• Heart rate at rest >110 bpm	• Reduction of systolic blood pressure during exercise (>10 mmHg)	
• Peak VO_2_ < 10 mL/kg/min	• Increasing ventricular or supraventricular arrhythmias	
• Ventricular tachycardia upon increasing exertion	• Vegetative reactions such as dizziness or nausea	
• Poorly controlled diabetes mellitus (in consultation with patient’s internal medicine specialist)		• Pulmonary problems No desaturation; this usually means that O2 saturation (SaO2) should remain ≥90 % during exercising (and should not fall by ≥4 %)^+^
• Fever		
• Acute systemic diseases		
• Recent pulmonary embolism (<3 months ago) causing severe haemodynamic strain		
• Thrombophlebitis		
• Acute pericarditis or myocarditis		
• Haemodynamically serious aortic stenosis or mitral valve stenosis		
• Heart valve failure constituting an indication for surgical intervention		
• Myocardial infarction less than 3 weeks before the start of the training		
• Atrial fibrillation with rapid ventricular response at rest (>100 bpm)		
• Serious cognitive problems (memory, attention and concentration)		
• Weight gain of >3 kg within a few days, whether or not accompanied by increased dyspnoea at rest		

^a^Symmetrical functional movements below the patient’s pain threshold (with comfortable rather than forceful movements and controlled breathing) can be started within 6 weeks after surgery (which can also help to prevent the development of a frozen shoulder)

^+^The physiotherapist should consult the patient’s pulmonologist or cardiologist to decide on the minimum individual saturation value

**Fig. 2:**
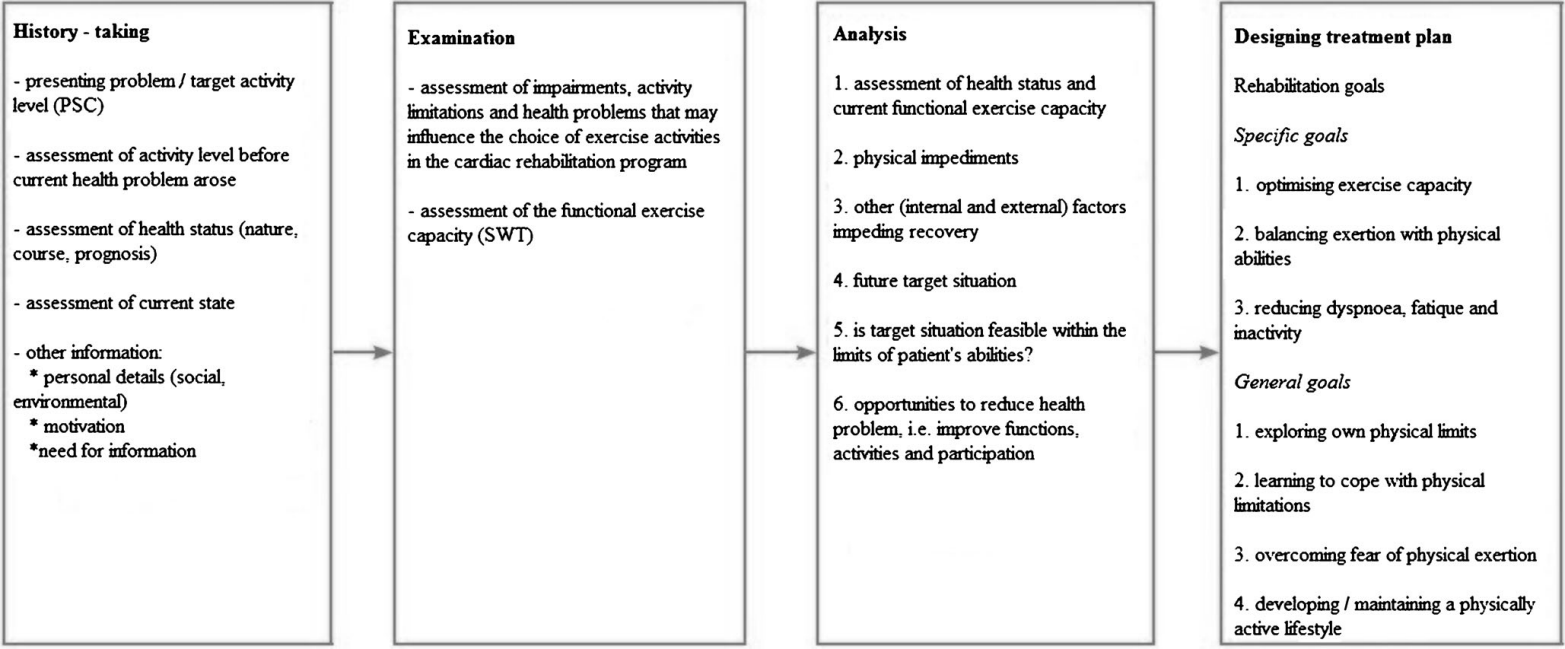
Flowchart of the assessment procedure performed by the physiotherapist

The aim of the physiotherapist’s assessment is to assess the nature and severity of patients’ health problems in relation to their physical functioning (in terms of movements) and to assess the extent to which this can be modified. Fig. 2 shows a flowchart of the assessment procedure.

The assessment focuses on identifying impairments of bodily functions, limitations of activities, restrictions of participation and health problems that may influence the choice of exercise activities to be included in the training program. Limitations of activities may regard their nature, duration and/or quality. The physiotherapist analyses the performance of problematic activities that were identified using the patient-specific complaints instrument. [23] The physiotherapist assesses the quality of the patient’s aspects of physical performance (including endurance, strength, speed, agility and coordination) and the degree to which the patient is able to use them. The physical performance during activities perceived as problematical can be scored in terms of duration and intensity, perceived fatigue (Borg Rating of Perceived Exertion (RPE) scale 6–20) [24] and in terms of anxiety, chest pain and dyspnoea (Borg 1–10). If requested by the patient’s physician, the physiotherapist can monitor the patient’s heart rate and blood pressure during these activities. The modified Shuttle Walk Test (SWT) [25–27] is used to determine patients’ functional exercise capacity. The MET method and the Specific Activity Scale (SAS) [28] can be used to estimate whether any discrepancy between the actual performance level and the target level can be eliminated with a suitable training program. The physiotherapist measures patients’ maximum inspiratory pressure (Pimax) using a Pimax meter. Based on the results of the assessment procedure, rehabilitation goals will be defined.

#### Treatment phase

**Fig. 3:**
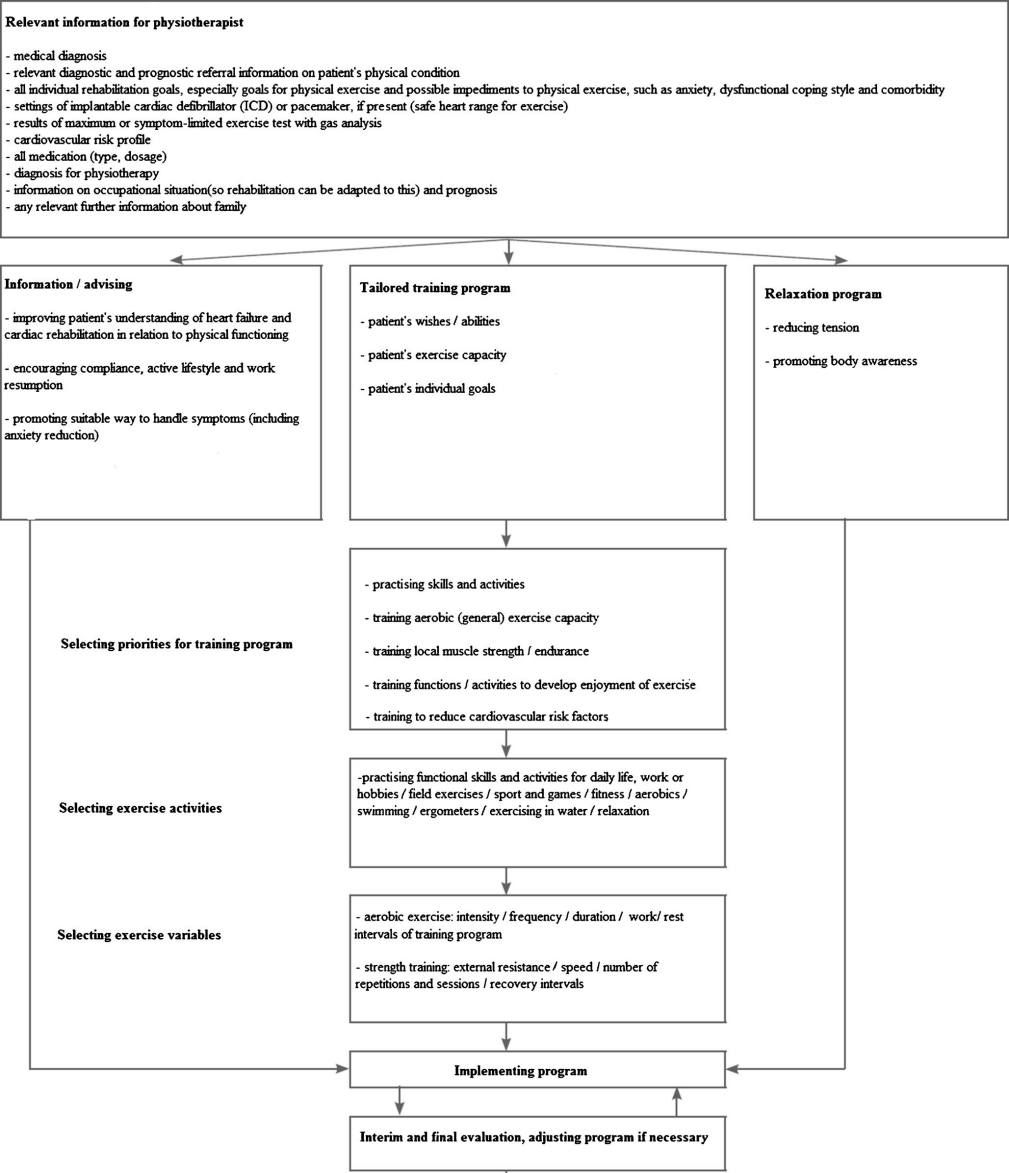
Flowchart of the physiotherapy process

The treatment during the outpatient CR phase comprises three modalities: information/advice, a tailored training program and a relaxation program (Fig. 3). The physiotherapist systematically evaluates the rehabilitation goals, during and at the end of the treatment. Typically, the treatment phase should last for a period 8 to 12 weeks in order to obtain the optimal treatment result.

### Information / advice

Information and advice must be given in a multidisciplinary context. The physiotherapist offers the patient assistance (guidance, coaching), information and advice, geared towards their personal goals. Patient education about the disorder and the importance of treatment are required to enable the patient to cope effectively with CHF.

Aims of information and advice may include:

- Improving patients’ understanding of their disorder, CR and illness beliefs;
- Education on a healthy active lifestyle;
- Recognising signs of deterioration (decompensation) of the CHF;
- Promoting compliance;
- Promoting effective ways of dealing with symptoms and exertion in daily life (level of dyspnoea and fatigue);
- Promoting return to work / occupational activities (where applicable, most patients are past retirement age).

### Tailored training program

The training program is intended for patients who are:

- Referred by a cardiologist and have no contraindications for training (Table 3);
- Functionally stable (i.e. no change in NYHA class) and on optimal medication for at least 3 weeks;
- NYHA Class II or III;

Based on the individual goals, patients’ preferences and limitations established during the assessment procedure in combination with results of the maximum or symptom-limited exercise test with respiratory gas analysis and safety criteria (Table 3), a definite training program is composed. It is important to realise that a substantial number of CHF patients do not respond to training in terms of an increase in VO_2_ peak [29, 30].

The physiotherapist observes the patient’s training intensity, individual response, tolerance of the exercise load and their overall clinical status. Also the patient’s response during the recovery phase after exercise is monitored. The exercise session must be terminated when safety criteria are exceeded, or if there are any reasons for excessive strain (Table 3).

During the first 2–4 weeks of the training program, the physiotherapist systematically measures the patient’s blood pressure and heart rate (and rhythm) before, during and after the training session. This supervised period is extended if any arrhythmias, ischaemia, angina, blood pressure abnormalities or supraventricular or ventricular ectopy occur during exercising. Patients with documented ischaemia or arrhythmias may need to have their cardiac rhythm monitored by ECG, if indicated by their cardiologist. In the case of comorbidities, the GDG recommends starting the training program, based on the exercise principles, relating to the exercise limiting factor, and/ or the most restrictive pathology or disorder. A low-intensity start is recommended in case of doubt.

The tailored training program may comprise practising skills and activities (to enable patients to utilise their general or strength endurance in motor activities), aerobic interval/ endurance training, strength endurance training (inspiratory muscle training and peripheral muscle training), practising functions/activities, and/or (aerobic) training to reduce cardiovascular risk factors (if atherosclerosis is the underlying case of CHF).

#### Recommendation 2. Aerobic endurance and/or interval training

Aerobic endurance or interval training increases the exercise capacity and QoL in patients with CHF (NYHA Classes II-III) and is therefore recommended. The mechanisms underlying these beneficial effects involve improvement of patients’ muscle perfusion, muscle metabolism, breathing efficiency, neurohormonal regulation and cardiac pump function (Level 1) [4–6].

It is assumed that high-intensity interval training (HIT) results in a better improvement of left ventricular function than moderate-intensity training (Level 2) [31].

HIT may result in a greater improvement of the aerobic endurance capacity than moderate-intensity training (Level 2) [32].

The research concerning the effectiveness of HIT training is conducted in relatively low-risk CHF patients; therefore the GDG advises to be cautious in patients with a high risk of cardiac overload (Level 4). If HIT is applied, the cardiologist should be informed and safety criteria (Table 3) should be closely adhered to.

Training should be individually directed and functionally geared toward personal goals. If the goal is to improve endurance capacity, aerobic exercise can be gradually increased from 50 to 80 % of VO_2_ peak/ heart rate reserve, preceded by warming up and followed by cooling down. HIT can involve interval blocks of 4 times 4 min at 80–90 % of VO_2_ peak / heart rate reserve, with active recovery for 3 min at 40–50 % of VO_2_ peak / heart rate reserve (as determined by the maximum or symptom-limited exercise test with respiratory gas analysis). During both endurance or interval training and HIT, the program should preferably start with a 2-week introductory period in which the patient trains at an intensity of 40–50 % of VO_2_ peak / heart rate reserve. Patients with a VO_2_ peak >10.5 mL/kg/min, but <17.5 mL/kg/min (3–5 METs/40–80 W) appear to benefit most from 1 to 2 training sessions a day for 15 min, focusing on aerobic interval training. Patients with a VO_2_ peak >17.5 mL/kg/min (≥5 METs / ≥80 W) can limit their training to 2–3 sessions a week, for 20–30 min per training session [9].

If the goal is to improve patients’ endurance capacity, training intensity should be based on the results of a maximum or symptom-limited exercise test with respiratory gas analysis (Table 4), preferably on a percentage of VO_2_ peak, VO_2_ reserve (the difference between the VO_2_ max and the VO_2_ at rest) or the ventilatory or anaerobic threshold, converted into heart rate (or work rate, Watt). If no respiratory gas analysis has been done, the maximum heart rate attained can be used to calculate the training zone. In both cases, the Karvonen formula is used to calculate the training heart rate as a percentage of the heart rate reserve, added to the resting heart rate. [33] If the patient’s heart rate does not rise sufficiently during the maximum or symptom-limited exercise test with respiratory gas analysis, the training intensity should be based on a percentage of the maximum capacity expressed in Power (Watt) or METs, and/or the Borg score (6–20).

**Table 4:** Information to determine training intensity

• The patient’s current physical condition, based on the maximum or symptom-limited exercise test with gas analysis (spiro-ergometry)
• Protocol used
• The referring physician’s evaluation of the electrocardiogram before, during and after exercise (criteria for cardiac ischaemia, arrhythmias and the practical consequences of the findings)
• Heart rate at rest, the maximum heart rate and recovery heart rate (especially during the first minute)
• Maximum VO2max and wattage achieved (and the percentage of the predicted value)
• Blood pressure changes at rest, during exercise and during the recovery phase
• The reason for terminating the test and the level of the impairment (central or peripheral)
• Medication use (type and dosage)
• The patient’s subjective symptoms during the test (angina/ dyspnoea) and his/her Borg score
• Spiro-ergometry: gas exchange parameters such as maximum oxygen uptake (VO2max), the percentage of predicted VO2max, O2 pulse, maximum respiratory minute volume (VE) (tidal volume and respiratory rate), respiratory exchange rate, anaerobic or ventilatory threshold, VE/VCO2 ratio, saturation and any other relevant parameters (e.g. VO2 oxygen uptake efficiency slope and the presence of respiratory oscillations)
• The maximum voluntary ventilation, which may be derived (37.5× the forced expiratory volume (FEV1))

#### Recommendation 3. Submaximal strength training

Strength training increases muscle strength and endurance, and is recommended in preparation for, or as an adjunct to, aerobic exercise training for patients with stable CHF (Level 1) [5, 34].

This type of exercise training is particularly suitable for patients who experience strength-related limitations in activities of daily living and during social participation. The GDG advises caution with strength training in the CHF patient (research has only been conducted in relatively low-risk CHF patients), especially in patients with a left ventricular function <35 % (Level 4).

Strength training should be functional and directed toward personal goals and individual restrictions in daily life. The strength training starts with a 2-week ‘pre-training’ period, involving 2–3 series of 10 repetitions against a low resistance estimated at <30 % of 1 repetition maximum (RM). After this pre-training period, the resistance level for strength training can be estimated on the basis of 10 RM. If the goal is to improve the patient’s muscle strength, the external resistance can be gradually raised from 40 to 65 % of the 1RM. Training the large muscle groups is recommended, at a frequency of 2–3 times a week, in 10–15 repetitions of 2–3 series.

#### Recommendation 4. Inspiratory muscle training (IMT)

IMT increases Pimax and reduces the sensation of dyspnoea and is therefore recommended in CHF patients with a Pimax <70 %predicted (Level 2) [35–37] or a ventilatory impairment according to the maximum or symptom-limited exercise test with respiratory gas analysis (i.e. insufficient or absent breathing reserve) (Level 4).

High-intensity IMT may produce better results than low-intensity IMT, however in practice the high load training is not suitable for patients with dyspnoea during low ADL effort (Level 4).

Low-intensity IMT should be performed against a resistance of 20–40 % of Pimax for 30 min/day or 2 times 15 min/day, on 3–4 days a week, preferably for a period of 8–12 consecutive weeks, and high-intensity IMT against a resistance of 60–70 % of Pimax for 4–5 times 5–10 min/day, 3–4 days a week, preferably 10 consecutive weeks, using a threshold device.

### Relaxation program

#### Recommendation 5. Relaxation therapy

A relaxation program (including breathing therapy) is recommended in CHF patients. A relaxation program can lead to tranquillity (a more quiet / less stressful mind), better breathing control (more regular breathing), reduced sensation of dyspnoea and an improvement of QoL (stress reduction) (Level 2) [38–48].

A relaxation program in combination with aerobic training is superior to training alone (Level 3) [40].

The CHF patient should attend two sessions to try out the relaxation program. If the program proves beneficial, they attend a further 6–8 sessions lasting 60–90 min each. An important goal of the relaxation program is to teach the patient to calm their mind and to breathe more slowly. In addition, the program may address cognitive themes such as understanding the value of rest, the balance between work and rest, the influence of psychological factors on physical functioning and differentiating between cardiac factors in relation to stress, anger, depression and pressure of time. Instructions for relaxation can be given during exercising (active relaxation) or at rest (passive relaxation), partly in the context of warming up and cooling down, and partly as a separate relaxation program.

#### Evaluation

In addition to a ‘continuous’ evaluation over the entire course of the training program, more comprehensive interim evaluations should be carried out at least every 4 weeks, as well as at the end of the CR program. Final evaluation criteria that may prevail are listed in Table 5.

**Table 5:** Situations that may prevail in the final evaluation at the conclusion of the CR program^a^

• The patient has attained his/her goals at an optimum level
• The patient has partially attained his/her goals, and it seems likely that he/she will be able to continue the training activities elsewhere, under supervision, and thus eventually attain these goals
• The patient has not attained his/her goals and it seems likely that he/she has attained their maximum achievable level. Not all patients will improve their endurance capacity and therefore need for example to spread their energy expenditure and to deal with their dyspnoea in a functional way

^a^After the outpatient CR phase, all patients with CHF are referred to after-care activities (Phase III); in exceptional cases they may be referred to a specialised CR centre for clinical rehabilitation

Table 6 shows the intended outcomes for the CR goals, the relaxation program and patients’ acquired knowledge about CHF and lifestyle, as well as recommendations for assessment and evaluation.

**Table 6:** Evaluation and screening instruments for each goal in physical therapy

Goal	Evaluation instrument	When	Final outcome
***Specific physical goals***			
I. Optimising exercise capacity	**By physician** • Maximum or symptom-limited exercise test with gas analysis plus Borg RPE scale (6–20), and as desired scoring Anxiety, Angina and/or Dyspnoea	At start and end of CR and / or training program	Exercise capacity is at optimum or target level for this patient
	**By cardiac rehabilitation coordinator** • Subjective physical score on KVL-H questionnaire		
	**By physiotherapist** • As for goals one and two • (modified) SWT • Possibly MET method and/or SAS	At start, every 4 weeks and at end of CR and/ or training program	Functional exercise capacity is at optimum or target level
II. Balancing exertion with physical abilities	• Compare subjective exercise capacity score with objective score • Ask about five most problematic activities (PSC) and score these on the Borg RPE scale (6–20); possibly score anxiety and/or angina and/or dyspnoea	At start and end of CR and / or training program, but also continuous evaluation to check for excessive strain	Patient (and partner) coping effectively with symptoms, that is, patient avoids excessive strain and (if possible) improves exercise capacity (goal one). Patient is able to spread his/her energy expenditure and to deal with the dyspnoea in a functional way
III. Reducing fatigue, dyspnoea and inactivity	• Borg RPE scale (6–20) for fatigue and dyspnoea • Monitor Movement and Health (www.tno.nl) (in Dutch)	At start and end of CR and / or training program	Patient’s sensation of fatigue and dyspnoea is at optimum or target level. Patient has adopted a physically active lifestyle
***General physical goals***			
1. Exploring one’s own physical limits	• Ask for five most problematic activities (PSC) • Ask patient to carry out problematic activities and possibly score them for duration and quality, perceived fatigue (Borg RPE 6–20) and in terms of anxiety and / or Angina and / or Dyspnoea (if indicated). • Monitoring heart rate and blood pressure	At start and end of CR and / or training program	Patient is aware of his/her own physical limits, i.e. knows what level of exertion is possible
2. Learning to cope with physical limitations		Monitoring heart rate, measuring blood pressure and scoring on Borg scale before, during and after each session	Patient can cope with physical limitations and utilise his/her limited energy efficiently, and has achieved a balance between exertion and relaxation
3. Overcoming fear of physical exertion	• History-taking and observation • Questionnaire: see Multidisciplinary Guideline CR 2011 (www.nvvc.nl) (in Dutch)	At start and end of CR and / or training program	Patient is no longer afraid of exertion
4. Developing an active lifestyle	• History-taking (motivational interviewing) • Monitor Movement and Health (www.tno.nl) (in Dutch) • Post-CR activities started	At start and at end of CR and / or training program	Patient has adopted an active lifestyle or is able to keep up the most active achievable lifestyle
***Focal points***			
Acquiring information about secondary prevention	• Checklist for risk factors / unhealthy behaviour • Phase III activities started • Ability to cope effectively with symptoms • Ability to recognise signs of decompensation	At start and at end of rehabilitation and / or training program	Patient knows about healthy living and secondary prevention
Goals of relaxation program	• Evaluation list • Using a flowchart	At interim and final evaluation of CR and / or relaxation program	Patient is familiar with the relaxation program and is able to relax

*Borg RPE scale* Borg Rating of Perceived Exertion, *KVL*-*H* Dutch quality of life questionnaire for heart patients, *6MWT* 6-min walking test, *MET* metabolic equivalent of task, *PSC* Patient-specific complaints, *SAS* Specific activity scale, *SWT* Shuttle walk test, *CR* cardiac rehabilitation

The physiotherapist should report to the multidisciplinary CR team about the treatment process, the treatment outcomes and the recommendations (aftercare). This should happen at least at the end of the treatment, but preferably also during the treatment period. In addition, the physiotherapist informs the patient’s cardiologist, family physician and, if applicable, their rehabilitation physician or company doctor.

### Post-CR phase (phase III)

#### Recommendation 6. Continuation of a physically active lifestyle

Patients are recommended to continue exercise, as part of an active lifestyle, for the rest of their lives after the outpatient CR period has ended, at a physiotherapy practice, at a certified exercise facility or independently (Level 1–2) [49–51].

Monitoring by secondary care professionals to check if CHF patients maintain their exercise capacity and an active lifestyle in order to identify relapses at an early stage and intervene is advisable (Level 3) [52].

Patients with an indication to attend high-intensity maintenance training (≥60 % VO_2_ peak) should be referred to a physiotherapy practice or a certified exercise facility (registered with the Dutch cardiac association), where professional supervision is available. Patients should preferably continue their training activities in a setting that participates in a local network which includes the hospital or rehabilitation centre where the CR program took place, as this implies easy access and frequent contacts. Patients requiring low- or moderate-intensity maintenance training (<60 % of VO_2_ peak) can choose to do this independently, or at a certified exercise facility. If patients who are advised to attend low- to moderate-intensity maintenance training are deemed likely to soon relapse into an inactive lifestyle, they should be referred to a training program at a primary care physiotherapy practice, under professional supervision.

## Applicability and implementation issues

This guideline outlines best practice standards for physiotherapy, in terms of efficacy, efficiency and tailored care, for CHF patients who are eligible for CR. Implementation of the guideline in clinical practice needs further evaluation. [53] Adherence to the guideline needs to be stimulated by, for example, adopting it into a decision supporting system/ flowchart, for example the Dutch clinical algorithm for patient needs in CR [22].

## Conclusion

Strong evidence is found for exercise-based CR in CHF patients, especially for aerobic exercise training (endurance, interval and HIT) during the outpatient rehabilitation, [4–6] and adopting training after supervised CR. [49–51] It can be assumed that treatment of pulmonary symptoms during the stay at the ICU/CCU (if necessary) and early mobilisation in the clinical phase (if applicable) leads to a faster recovery, [20, 21] and also that strength training, [5, 34] inspiration muscle training (in case Pimax <70 %predicted or when the patient has ventilatory impairment) [35–37] and a relaxation program [38–48] are effective in increasing Qol and exercise capacity, particularly in combination with aerobic exercise training.

This guideline is the first guideline for physiotherapists that provides practical guidance on how to tailor an exercise training program with respect to intensity and duration individually, using results of a maximum or symptom-limited exercise test with respiratory gas analysis. This guideline and also the guideline for exercise-based CR in patients with coronary artery disease [15] aims to reduce the considerable practice variation which has recently been reported in Dutch CR centres, [7] and thereby, to increase quality of exercise-based CR in the Netherlands [54].

Further research is needed on strategies to improve monitoring and follow-up of the maintenance of a physically active lifestyle after supervised CR; for example by implementing activity monitoring devices combined with telemonitoring, or by web-based coaching platforms to guide patients. [55] Exercise-based CR may also be followed by relatively brief maintenance programs and booster sessions, including behavioural techniques and focusing on incorporating lifestyle changes into daily life, in order to improve long-term adherence to lifestyle modifications. [56] Finally, more research is needed into characteristics and modalities of physical activity and exercise training in CHF in the long term.
